# Distinguishing Benign Notochordal Cell Tumors From Chordoma in a Patient With Multicentric Spinal Lesions

**DOI:** 10.1155/crra/6604031

**Published:** 2026-05-12

**Authors:** Elleana A. Paradise, Justin A. Schmidgall, David S. Baskin, Robert E. Jackson, Bin S. Teh, Steve H. Fung

**Affiliations:** ^1^ School of Engineering Medicine, Texas A&M University, Houston, Texas, USA, tamu.edu; ^2^ Department of Radiology, Houston Methodist Hospital, Houston, Texas, USA, houstonmethodist.org; ^3^ Department of Neurosurgery, Houston Methodist Hospital, Houston, Texas, USA, houstonmethodist.org; ^4^ Weill Cornell Medical College, New York, New York, USA, cornell.edu; ^5^ Naresh K. Vashisht College of Medicine, Texas A&M University, Bryan, Texas, USA, tamu.edu; ^6^ Houston Methodist Research Institute, Houston, Texas, USA, houstonmethodist.org; ^7^ Kenneth R. Peak Brain and Pituitary Treatment Center, Houston Methodist Hospital, Houston, Texas, USA, houstonmethodist.org; ^8^ Department of Medicine, Houston Methodist Hospital, Houston, Texas, USA, houstonmethodist.org; ^9^ Department of Radiation Oncology, Houston Methodist Hospital, Houston, Texas, USA, houstonmethodist.org

**Keywords:** atypical notochordal cell tumor, benign notochordal cell tumor (BNCT), chordoma, multicentric

## Abstract

This case report details a 60‐year‐old man who presented with several spinal lesions after they were discovered incidentally during abdominal CT imaging. Lesions were observed in the cervical and thoracic spine and sacrum (C2‐C3, C3‐C4, C5‐C6, T3, T4, T8‐T9, S2‐S3, and S4 levels) with varying bone involvement, soft tissue extension, and appearance on CT and MRI. The dominant lytic T8–T9 lesion was biopsied, resulting in a diagnosis of chordoma, yet ^18^F‐FDG PET/CT showed no significant FDG activity in this lesion or elsewhere. Other lesions were not biopsied, and all lesions were subsequently monitored with routine imaging. Over the next several years, the lesions continued to show long‐term stability on MRI with no symptomatic progression. Due to their longstanding benign nature, the unbiopsied lesions were reevaluated as a more benign etiology, such as benign notochordal cell tumors (BNCTs), which are also derived from notochordal remnants and are hypothesized as a precursor lesion to chordomas, but differ in their clinical behavior, histopathological characteristics, and imaging features. Accurate differentiation between BNCTs and chordomas is imperative for guiding treatment strategies, especially in the very rare case of multiple confounding lesions, as seen in this patient. This report underscores the diagnostic challenges in differentiating BNCTs and chordomas and highlights the importance of correlating histopathological findings with clinical and imaging features for accurate interpretation and intervention.

## 1. Introduction

Benign notochordal cell tumors (BNCTs) and chordomas are both derived from notochordal remnants but differ in their clinical behavior, histopathological characteristics, and imaging features [1–3]. Distinguishing between these entities is crucial for appropriate patient management, as chordomas are typically malignant and require aggressive treatment, whereas BNCTs are benign and often require only observation [4]. It is hypothesized that BNCTs may be a precursor to chordoma, appearing benign but eventually progressing into malignant, locally aggressive lesions, furthering the complexity of the relationship between these pathologies [1, 5–7].

## 2. Case Presentation

A 60‐year‐old man presented to our medical center for evaluation of multiple spinal lesions. The patient was asymptomatic and had a prior history of smoking (1 pack/day for 15 years; quit 20 years ago). His clinical history began 2 years prior when he developed colitis and underwent abdominal CT for assessment, which incidentally revealed multiple lesions in the thoracic spine, including a large, lytic lesion at the T8–T9 vertebrae.

About 2 years later, the patient underwent a needle biopsy of the T8–T9 lesion at another facility. Histology showed nests and lobules of cells with rounded nuclei and abundant eosinophilic vacuolated cytoplasm, and a myxoid background. Immunohistochemical stains were positive for brachyury, cytokeratin (AE1/AE3), S‐100, and CK7, and negative for PAXB, CK20, TTF1, Melan A, arginase, desmin, and SMA. There was cytoplasmic reactivity for SOX10. These findings supported a diagnosis of chordoma for the T8–T9 lesion. However, ^18^F‐FDG PET/CT showed no significant FDG activity within the lytic lesion or elsewhere.

**Figure 1:**
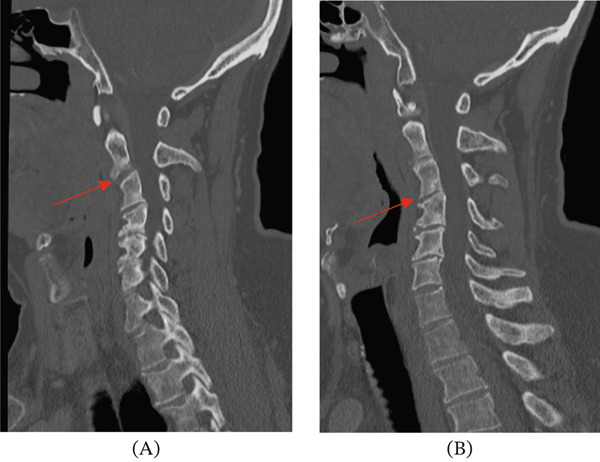
These CT scans of the cervical spine in our patient show that the (A) right C2–C3 uncovertebral joint (red arrow) and (B) left C3–C4 uncovertebral joint (red arrow) lesions are poorly visualized on CT, showing no significant sclerotic or lytic features.

**Figure 2:**
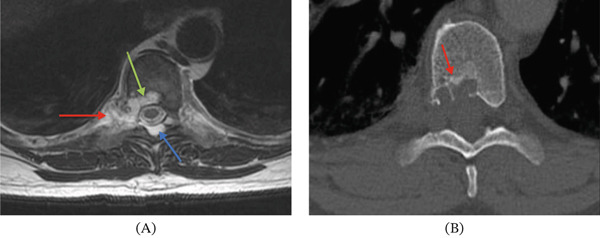
These axial images of the thoracic spine show the dominant T8–T9 lesion, which was diagnosed as chordoma after needle biopsy. (A) Panel A shows a lobulated T2‐hyperintense mass with well‐defined margins involving bone (green arrow), with paraspinal (red arrow) and epidural (blue arrow) extension on MRI. (B) Panel B shows a lytic appearance on CT with sclerotic borders (red arrow). These features are radiologically consistent with chordoma.

**Figure 3:**
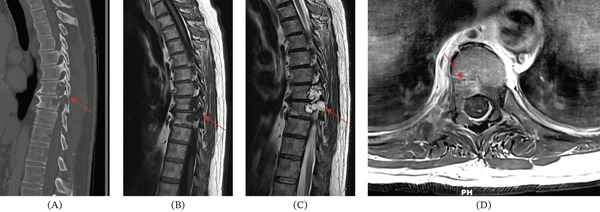
These sagittal and axial images further show the dominant T8–T9 lesion. (A) Panel A shows a lytic appearance on CT (red arrow). (B) Panel B shows low signal on noncontrast T1 MRI (red arrow). (C) Panel C shows T2 hyperintensity (red arrow). (D) Panel D shows moderate contrast enhancement in the anterior portion of the lesion (red arrow). These features are radiologically consistent with chordoma.

**Figure 4:**
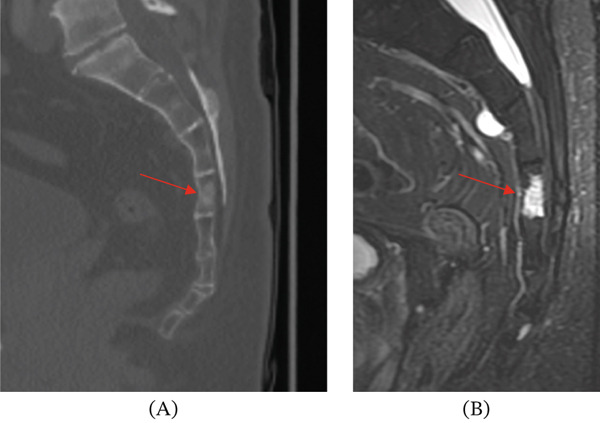
These images show the sacral lesions in our patient at S2–S3 and S4. (A) Panel A shows an intraosseous sclerotic lesion at S4 (red arrow) on sagittal CT. (B) Panel B shows the S4 lesion (red arrow) with a high signal on T2 STIR. These features at S4 are more typical of a BNCT.

When the patient presented to our medical center for further evaluation, additional imaging was performed. Cervical spinal CT revealed spondylosis but no definitive lytic or sclerotic bony lesion or gross soft tissue mass (Figure 1). Thoracic spinal CT showed a lytic lesion with sclerotic margins involving the right posterolateral aspect of the T8 and T9 vertebral bodies, right pedicles, and extraosseous extension to the adjacent paraspinal and epidural spaces and right neural foramen (Figures 2 and 3). A similar but smaller lytic lesion with sclerotic margins was observed involving the T4 left lateral vertebral body and pedicle with extraosseous extension to the left T3–T4 neural foramen. Lumbar spinal CT showed a soft tissue density presacral lesion at S2–S3 without significant bone involvement and a sclerotic intraosseous lesion at S4 (Figure 4).

**Figure 5:**
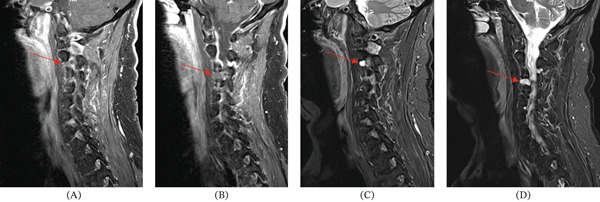
These MRI sequences of the cervical spine in our patient show T2‐hyperintense, nonenhancing lesions at the right C2–C3 uncovertebral joint region and left C3–C4 uncovertebral joint region. (A,B) Panels A and B are T1‐weighted fat‐saturated images showing the lesions (red arrows) at C2–C3 and C3–C4, respectively. (C,D) Panels C and D are T2‐weighted STIR images showing the lesions (red arrows) at C2–C3 and C3–C4, respectively.

Cervical spinal MRI revealed small bone lesions involving the right C2–C3, left C3–C4, and left C5–C6 uncovertebral joint regions (Figure 5) that were not well visualized on CT (Figure 1). These lesions were T2 hyperintense and nonenhancing with smooth margins and minimal cortical bone involvement. Thoracic spinal MRI revealed a small T2 hyperintense lesion involving the T3 lateral vertebral body, primarily extraosseous and not well visualized on CT. The larger lytic lesions on CT involving the T4 and T8–T9 levels described above were T2 hyperintense and moderately enhancing on MRI (Figures 2 and 3). Sacral MRI showed a T2 hyperintense presacral lesion with periosteal involvement at S2–S3 with smooth margins and no definitive bone involvement, and a separate T2 STIR hyperintense intraosseous lesion corresponding to the sclerotic lesion at S4 (Figure 4), both showing no to minimal enhancement on postcontrast images.

We have continued to monitor this patient with routine imaging over 7+ years since the lesions were discovered and the lesions have not shown any progression or malignancy, nor has the patient reported any symptoms. In the sixth year of monitoring, we noted a possible minor increase in size (1 mm) in the right paraspinal and epidural component of the T8–T9 lesion. Due to their benign nature and lack of symptoms, no further treatment or surgery has been pursued for any of the lesions. We will continue to observe the patient on an annual basis.

**Figure 6:**
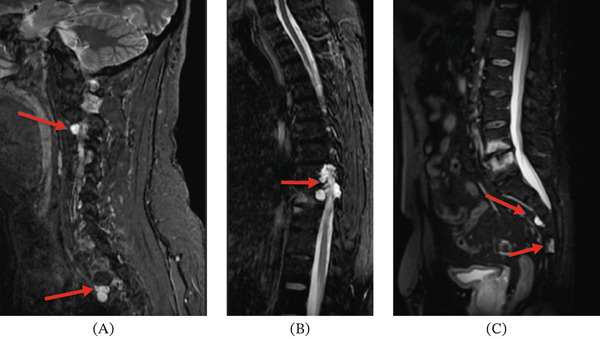
These images on sagittal STIR show the widespread spinal lesions in the patient. (A) Panel A shows T2‐hyperintense lesions in the cervical and upper thoracic spine at C2–C3, T3, and T4 (red arrows). (B) Panel B shows T2‐hyperintense lesion in the thoracic spine at T8–T9 (red arrow). (C) Panel C shows T2‐hyperintense lesions in the sacral spine at S2–S3 and S4 (red arrows)

Figure 6 includes STIR imaging of the spectrum of lesions in the cervical, thoracic, and sacral spine. A summary of the observed lesions is included in Table 1 below.

**Table 1:** Summary of lesion findings on repeat imaging studies.

Level	Location	T1 signal	T2 signal	Enhancement	CT appearance
C2–3	Right uncovertebral joint region	Low	High	Nonenhancing	Not well visualized
C3–4	Left uncovertebral joint region	Low	High	Nonenhancing	Not well visualized
C5–6	Left uncovertebral joint region	Low	High	Nonenhancing	Not well visualized
T3	Left lateral vertebral body; primarily extraosseous with minimal intraosseous component	Low	High	Nonenhancing	Soft tissue density similar to fluid
T4	Left T4 pedicle and extraosseous extension into the left T3‐4 neural foramen	Low	High	Enhancing	Lytic
T8–9	Confluent lesion involving right T8 and T9 posterolateral vertebral bodies, right pedicles, and laminae; extraosseous extension into epidural space and right T8–9 neural foramen	Low	High	Enhancing	Lytic
S2–3	Midline presacral; primarily extraosseous with no significant intraosseous component	Low	High	No to minimal enhancement	Soft tissue density, similar to fluid
S4	Midline intraosseous lesion involving S4 vertebral body	Low	High	No to minimal enhancement	Sclerotic

## 3. Discussion

Due to some of their similar and sometimes overlapping radiologic and histologic characteristics, BNCTs and chordomas are often difficult to distinguish, especially in cases with multiple lesions, as seen in this patient. BNCTs and chordomas are both derived from notochordal remnants but differ in their clinical behavior, histopathological characteristics, and imaging features [1–3]. In fact, multiple studies have posited that BNCTs and chordomas are related and that BNCTs may be a precursor lesion that can eventually undergo malignant transformation to become chordomas [1, 5–7], but this mechanism is not well understood. Furthermore, Carter et al. have documented “atypical” notochordal cell tumors (ANCTs) with radiologic and/or histologic features that do not meet diagnostic criteria for either BNCT or chordoma [4]. This case report adds to the literature on BNCTs and chordomas, and the importance of distinguishing the two entities.

This patient case was previously described as indolent multicentric chordoma [8]; however, after four additional years of observation and imaging, continued analysis of radiological findings and expanded review of the literature suggests that some of the intraosseous, nonenhancing lesions are more consistent with BNCTs rather than true chordoma.

Accurate differentiation between BNCTs and chordomas is imperative for guiding treatment strategies. Chordomas, due to their aggressive nature, require surgical resection with wide margins and subsequent radiotherapy, with a high potential for recurrence [4, 9, 10]. In contrast, BNCTs are benign and typically asymptomatic, with a low risk of progression; thus, they may be managed conservatively with regular monitoring [4, 10]. Misdiagnosis can lead to overtreatment of BNCTs or undertreatment of chordomas, both of which have significant implications for patient outcomes.

Histologically, BNCTs are characterized by sheets of vacuolated, physaliphorous cells with minimal nuclear atypia, lacking the lobular architecture and myxoid matrix typical of chordomas [1–3, 5, 10]. In contrast, chordomas exhibit cords or strands of atypical physaliphorous cells set within a prominent myxoid stroma [1, 5, 10], as observed in the T8–T9 biopsy in this patient. Both lesions may express markers such as cytokeratin, brachyury, and S‐100 protein [1, 11], which can complicate differentiation based solely on immunohistochemistry. Moreover, Shen et al. reported that fetal notochordal cell rests were completely negative for brachyury expression, whereas positive in chordomas [12]. It was also noted in six cases of classic chordoma with coexisting benign notochordal cell rests that brachyury was not expressed in the notochordal cell rests but was strongly expressed in the adjacent chordoma tumors [13]. As a result, it is thought that brachyury may play a role in tumorigenesis and progression [14, 15]. In our patient, the T8–T9 lesion stained positive for brachyury, cytokeratin (AE1/AE3), S‐100, and CK7, and negative for PAXB, CK20, TTF1, Melan A, arginase, desmin, and SMA. Other lesions in the cervical spine and sacrum were not biopsied.

Imaging also plays a pivotal role in differentiating BNCTs from chordomas. BNCTs typically present as intraosseous sclerotic lesions and are confined within the bone without cortical disruption or soft tissue extension [1, 2], but in rare cases may be extraosseous without soft tissue extension [2, 16] or may be associated with small soft tissue components on the periosteum [2]. Carter et al. reported three cases of tumors with subtle radiologic findings of cortical permeation and minimal soft tissue extension but histologic characteristics of BNCTs [4]. In our patient, the lesions had varying levels of soft tissue involvement. The dominant T8‐T9 lesion showed extraosseous extension into the epidural space, yet other lesions in the cervical spine and sacrum were intraosseous or exhibited possible minor cortical breakthrough.

**Figure 7:**
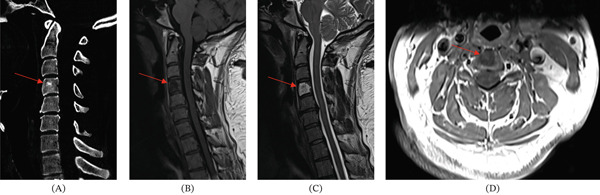
These images of the cervical spine are from another patient treated at our medical center with a likely BNCT diagnosis, showing typical BNCT radiological features for comparison. (A) Panel A shows an intraosseous sclerotic lesion (red arrow) on sagittal CT. (B) Panel B shows low signal on nonenhanced T1 MRI (red arrow). (C) Panel C shows T2 hyperintensity (red arrow). (D) Panel D shows no enhancement on postcontrast axial T1 (red arrow).

On CT scans, BNCTs typically appear as areas of sclerosis without bone destruction [16], while MRI reveals low‐to‐intermediate signal intensity on T1‐weighted images and high signal intensity on T2‐weighted images, with no to minimal enhancement after contrast administration [1, 2, 10, 16–18]. A relative lack of contrast enhancement is considered a hallmark of BNCTs; however, Kreshak et al. documented one case of a sclerotic BNCT with moderate contrast uptake and cystic appearance on T2‐weighted imaging [1]. Figure 7 includes CT and MRI imaging from another patient treated at our institution showing imaging characteristics typical of BNCTs.

In contrast, chordomas are typically osteolytic lesions that cause bone destruction and often extend beyond the osseous confines, forming a soft tissue mass [16]. On CT scans, chordomas present as lytic bone lesions with possible calcifications. MRI characteristics include low‐to‐intermediate signal intensity on T1‐weighted images and intermediate‐to‐high signal intensity on T2‐weighted images [16], which are not very distinct from BNCTs. However, unlike BNCTs, chordomas usually show some level of contrast enhancement [1, 10, 16].

Despite these distinguishing features, differentiating BNCTs from chordomas can be challenging, especially in cases where imaging and histopathological findings overlap. A review of the clinical, histochemical, and radiological findings in our patient highlights these nuances and offers a potential reinterpretation of some of the lesions as BNCTs. BNCTs are typically asymptomatic and exhibit long‐term stability on imaging [18], as observed in our patient over 7 years with no changes in lesion size or characteristics, other than slight (1 mm) growth in the T8–T9 lesion in the sixth year. Chordomas, by contrast, are locally aggressive and often show slow but progressive growth and symptomatic presentation [9, 10]. Our patient has not reported any symptoms (e.g., numbness, tingling, weakness) related to his numerous spinal lesions, which were found incidentally during an unrelated scan, suggesting the lesions have been present even longer than 7 years.

Furthermore, the multicentricity of lesions involving various levels of the spine and varying involvement of surrounding bone and soft tissue is intriguing, as chordomas are typically solitary and more likely to cause extensive soft tissue invasion and bone destruction [16]. Chordomas arising from multiple primary foci along the neuraxis, known as multicentric chordoma, are very rare with only a few cases reported, often showing clinical symptoms requiring surgical resection and radiotherapy [19–21]. Likewise, multicentric BNCTs are also a rare occurrence [3], although many cases may remain undetected due to their benign presentation. There have been documented cases of BNCTs and chordomas coexisting in the same patient, often discovered after histologic analysis of surgically resected chordoma lesions [5, 10, 13].

Some of the lesions in this patient, notably in the cervical and sacral spine, were sclerotic or not well seen on CT, and demonstrated predominantly smooth margins, minimal cortical bone involvement, and no soft tissue extension, whereas other lesions, notably in the thoracic spine, exhibited locally invasive behavior and were associated with cortical bone penetration and associated soft tissue extension and foraminal invasion (Table 1). Based on a review of the growing literature on BNCTs and chordomas, there are very few definitive findings to confirm a diagnosis in the absence of a thorough histopathological examination [14]. Although highly atypical of BNCTs, faint contrast enhancement and moderate soft tissue involvement may not rule them out [1, 2]. Many of the lesions in our patient exhibited criteria consistent with BNCTs, including sclerosis, lack of contrast enhancement, and little‐to‐no soft tissue involvement, suggesting a spectrum of benign tumors and malignant, yet stable, chordomas (or ANCTs, as described by Carter et al.), and further suggesting a potential relationship between BNCTs and chordomas.

This diagnosis is further supported by the ^18^F‐FDG PET/CT imaging for our patient. Few studies have used ^18^F‐FDG PET/CT in characterizing chordomas. One study that evaluated 23 patients with chordomas showed that all of the biopsy‐proven lesions had moderate heterogeneous FDG activity with an average SUVmax of 5.8 ± 3.7 and no statistically significant associations between FDG activity and MRI features [22]. Another study that compared 10 patients with sacral chordomas to 12 patients with other sacral malignancies showed the sacral chordoma lesions had significantly lower FDG activity compared with that of other malignancies [23]. Our patient had an ^18^F‐FDG PET/CT obtained after the initial diagnosis of chordoma by needle biopsy of the T8–T9 vertebral lesion, which showed no significant FDG uptake of the biopsied lesion or elsewhere.

When considering differential diagnosis of BNCT and chordoma, other tumors potentially involving the skull base and spine should be excluded, including chondrosarcoma, chondroid chordoma, giant cell tumor of bone, schwannoma, osseous metastasis, plasmacytoma, multiple myeloma, and other primary bone sarcomas. Of note, chondrosarcoma can be indistinguishable from chordoma on conventional imaging [24, 25]. Both have very high T2 signal and low to intermediate T1 signal and can have heterogeneous enhancement. Both can show areas of susceptibility changes on GRE and SWI due to hemorrhage in chordoma and mineralization and calcification in chondrosarcoma. Both have a predilection for the skull base with chordomas generally midline involving the clivus and chondrosarcoma off‐midline involving the petro‐occipital synchondrosis, although these features may overlap due to variable patterns of origin, growth, and invasion. Both lesions can involve the spine, with chordomas commonly involving the sacrococcygeal region and less frequently vertebral bodies (cervical > lumbar > thoracic), often extending across intervertebral disc spaces. Chondrosarcoma tends to involve the thoracic spine with posterior elements more frequent than vertebral bodies [26]. Chordomas, especially poorly‐differentiated chordomas, tend to have lower ADC values on DWI than chondrosarcomas, which generally have high ADC values [25, 27]. Histologically, brachyury immunostaining is the gold standard for chordoma, positive in 85%–98% of chordomas but negative in chondrosarcomas [25, 28, 29]. Additional markers include cytokeratin/EMA (positive in chordoma and negative in chondrosarcoma) and D2‐40 (positive in chondrosarcoma and negative in chordoma). Classic chordoma and variant chondroid chordoma both exhibit physaliphorous cells, but chondroid chordoma also has a cartilaginous component [24].

## 4. Conclusion

A thorough review of the literature suggests that our patient has a range of BNCTs and atypical chordomas based on available patient imaging (CT, MRI, and ^18^F‐FDG PET/CT) and needle biopsy of the T8–T9 lesion. All of the lesions have shown remarkable long‐term stability with a lack of clinical and symptomatic progression. This case underscores the diagnostic challenges in differentiating these entities and highlights the hypothesized relationship between the two entities given the multicentric yet benign nature of the lesions. It further highlights the importance of correlating histopathological findings with clinical and imaging features for accurate interpretation and intervention. Continued follow‐up with routine imaging is an appropriate management strategy for this patient, given the lack of symptoms or lesion growth. We will continue to evaluate this patient on a routine basis to observe the progression of these lesions.

## Funding

No funding was received for this manuscript.

## Conflicts of Interest

The authors declare no conflicts of interest.

## Data Availability

The data that support the findings of this study are available on request from the corresponding author. The data are not publicly available due to privacy or ethical restrictions.
